# FOP in South Africa: awareness leads to diagnosis

**DOI:** 10.1186/1546-0096-9-S1-P31

**Published:** 2011-09-14

**Authors:** Christiaan Scott

**Affiliations:** 1 Red Cross War Memorial Childrens Hospital, CAPE TOWN, South Africa

## Introduction

Fibrodysplasia Ossificans Progressiva (FOP) is a genetic disorder (autosomal dominant)in which there is progressive ossification of skeletal muscle. The majority of affected persons represent new mutations for the determinant gene, ACVR1 the protein product of which acts to regultae Bone Morphogenic Protein expression.(1)FOP presents in early childhood, with painful , hard areas of ossification in the muscles of the back/neck and progressive limitation leading to immbobilisation of all joints by adulthood.Previously there were few reports of FOP from Africa.We recently reported on 3 patients from South Africa(2) and have in the last 18months diagnosed a further 6 patients.

## Methods

We document 9 affected individuals in the Xhosa, Afrikaner and mixed ancestry community.

## Results

Two were reported in childhoodalmost three decades ago, five are young children, and two presented in their teens. All have the typical features of heterotopic ossification as well as the characteristic halluxvalgus. Two patients underwent unnecessary and harmful biopsies and one patient sustained a fracture of one of the areas of heterotopic bone during positioning for diagnostic xrays. In these three cases the tissue trauma exacerbated and stimulated heterotopic bone formation.

## Conclusion

FOP is a devastating condition with no known cure, but early diagnosis is essential to prevent unnecessary and directly harmful special investigations.In our experience an increased awareness has led to a flurry of new diagnoses, leading to early referral and appropriate counselling and management. Paediatric rheumatologists should be aware of this condition, in order to facilitate patient diagnosis and avoid harmful procedures.
